# Synthesis and Cytotoxic Activity of Biphenylurea Derivatives Containing Indolin-2-one Moieties

**DOI:** 10.3390/molecules21060762

**Published:** 2016-06-10

**Authors:** Wagdy M. Eldehna, Mohamed Fares, Hany S. Ibrahim, Muhammad A. Alsherbiny, Mohamed H. Aly, Hazem A. Ghabbour, Hatem A. Abdel-Aziz

**Affiliations:** 1 Department of Pharmaceutical Chemistry, Faculty of Pharmacy, Egyptian Russian University, Badr City, Cairo 11829, Egypt; ph.fares@yahoo.com (M.F.); hany.s.ibrahim@gmail.com (H.S.I.); 2 School of Chemistry, University of Wollongong, Wollongong 2522, New South Wales, Australia; 3 Department of Pharmacognosy, Faculty of Pharmacy, Cairo University, Kasr El-Aini Street, Cairo 11562, Egypt; Muhammad.alsherbiny@pharma.cu.edu.eg; 4 Department of Pharmacology and Toxicology, Faculty of Pharmacy, British University in Egypt, Cairo 11562, Egypt; mohamed.aly@bue.edu.eg; 5 Department of Biology, The American University in Cairo, New Cairo 11835, Egypt; 6 Department of Pharmaceutical Chemistry, College of Pharmacy, King Saud University, P.O. Box 2457, Riyadh 11451, Saudi Arabia; ghabbourh@yahoo.com; 7 Department of Applied Organic Chemistry, National Research Center, Dokki, Giza 12622, Egypt; hatem_741@yahoo.com

**Keywords:** biphenylurea, cytotoxic activity, indolinone, ADME

## Abstract

In our endeavor towards the development of potent anticancer agents, two different sets of biphenylurea-indolinone conjugates, **5a**–**s** and **8a**,**b** were synthesized. The *in vitro* cytotoxicity of the synthesized compounds was examined in two human cancer cell lines, namely MCF-7 breast cancer and PC-3 prostate cancer cells using the sulforhodamine B (SRB) colorimetric assay. In particular, the MCF-7 cancer cell line was more susceptible to the synthesized compounds. Compound **5o** (IC_50_ = 1.04 ± 0.10 μM) emerged as the most active member in this study against MCF-7, with 7-fold increased activity compared to the reference drug, doxorubicin (IC_50_ = 7.30 ± 0.84 μM). Compounds **5l**, **5q** and **8b** also exhibited superior cytotoxic activity against MCF-7 with IC_50_ values of 1.93 ± 0.17, 3.87 ± 0.31 and 4.66 ± 0.42 μM, respectively. All of the tested compounds were filtered according to the Lipinski and Veber rules and all of them passed the filters. Additionally, several ADME descriptors for the synthesized compounds **5a**–**s** and **8a**,**b** were predicted *via* a theoretical kinetic study performed using the Discovery Studio 2.5 software.

## 1. Introduction

According to the World Health Organization (WHO) estimates, cancer is a disease responsible for major morbidity and mortality worldwide. As reported in 2012 it resulted in about 8.2 million deaths [1]. Owning to its high incidence, prevalence, morbidity as well as mortality rates, cancer has been identified as a huge burden on the society. According to the statistics provided by GLOBOCAN in 2012, around 14.1 million newly diagnosed cancer cases were recorded. Among the newly diagnosed cases in females, breast cancer ranked in the first position worldwide and was regarded as the main cause of deaths attributed to cancer. On the other hand, prostate cancer was reported to be of the highest incidence among males in developed countries [2]. Although extensive research work had been conducted to synthesize and evaluate novel anticancer agents [3,4,5,6,7,8], satisfactory progress, relative to the speed of the disease progression, towards the development of anticancer agents of optimum activity and minimum side effects is not yet been accomplished.

**Figure 1:**
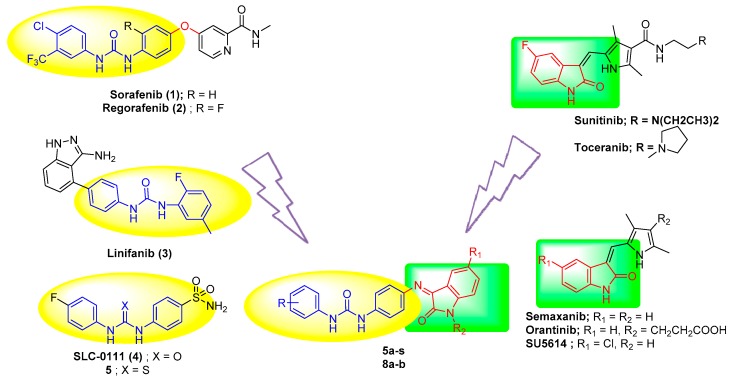
Biphenylurea derivatives and indolinone derivatives as anticancer agents and the proposed structures **5a**–**s** and **8a**,**b**.

Biphenylurea derivatives showed interesting activity as anticancer agents. For example, sorafenib, (**1**, Figure 1) is a potent diphenylurea VEGFR-2 inhibitor in addition to its inhibitory action on c-Kit, PDGFRβ, and Raf kinases (Figure 1) [9]. The U.S. FDA has approved it for the treatment of advanced renal cell carcinoma (RCC) and for hepatocellular carcinoma (HCC) [10]. Regorafenib (**2**, Figure 1), a fluoro derivative of sorafenib, [11] inhibits angiogenic kinases VEGFR-1/3, PDGFRβ, FGFR1, and Tie-2. Furthermore, it showed anti-proliferative activities on different cancer cell lines [12]. Regorafenib was approved for previously treated metastatic colorectal cancer (mCRC) and advanced gastrointestinal stromal tumors (GIST) [13]. Linifanib, (**3**, Figure 1), a novel biphenylurea derivative in phase II clinical trials, has selective inhibitory action on VEGFR and PDGFRs in patients with locally advanced or metastatic non-small cell lung cancer [14]. SLC-0111 (**4**) is a low molecular weight biphenylurea derivative in phase I clinical trials which has shown significant antitumor/ antimetastatic activity in animal models of the disease via the inhibition of tumor-associated human carbonic anhydrase (hCA) IX and XII isoforms (*Ki* = 4.5 and 45 nM, respectively) [15]. Its thioureido analog **5** displayed selective inhibition against tumor-associated hCA IX and hCA XII isoforms with *Ki* = 39, 4 nM, respectively [16].

On the other hand, the indolinone moiety proved its success in many compounds as antineoplastic [17,18]. This success is supported by the FDA approval of the use of indolinone derivative, sunitinib maleate (**20a**) (Sutent^®^) in combating advanced renal carcinoma [19] and gastrointestinal stromal tumors [20]. In addition, toceranib phosphate (**20b**) [21] (Palladia^®^), an orally bioavailable compound, is approved by FDA for the treatment of cutaneous (skin-based) mast cell tumors. On the other hand there are many compounds derived from indolinone are in clinical trials, for example, SU5416 (Semaxanib, **21a**) is an indolinone derivative in clinical trials, it showed extreme potency with GI_50_ values of <10 nM against 46 out of 53 NCI cell lines [22] but the clinical trials at phase III stopped due to its high toxicity [23]. Orantinib (**21b**) (SU6668), which is a more water soluble indolinone derivative than semaxanib, has significant anti-tumor activity in xenografts after daily oral treatment [24,25]. SU5614 (**21c**), in phase II clinical trials, induces growth arrest and apoptosis in c-kit expressing Kasumi-1, UT-7 and M-07e cells [26].

Incorporation of biphenylurea with an indolin-2-one moiety was proved as a successful strategy to get active hits to treat hepatocellular carcinoma and to affect cancer cells by inhibition of tumor-associated carbonic anhydrases [4,27]. As an extension of this work, new biphenylurea derivatives **5a**–**s** and **8a**,**b** (Figure 1) incorporating indolin-2-one moieties were investigated for their potential cytotoxic effect towards human breast (MCF-7) and prostate (PC-3) cancer cell lines. Moreover, physicochemical properties and prediction of ADME descriptors were employed to obtain useful data about their physicochemical interactions within the body.

## 2. Results and Discussion

### 2.1. Chemistry

**Scheme 1:**
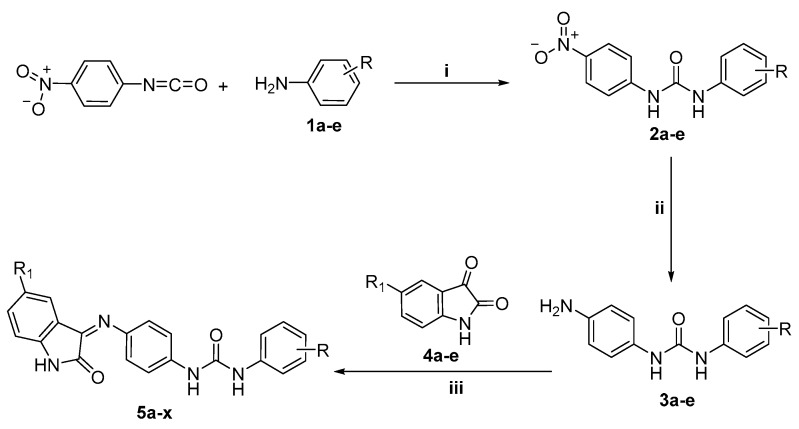
Synthesis of target compounds **5a–x**: *Reagents and conditions*: **i**, CH_3_CN, reflux 2h; **ii**, H_2_, Pd/C, MeOH, rt; **iii**, EtOH, AcOH (catalytic), reflux 3 h.

**Scheme 2:**
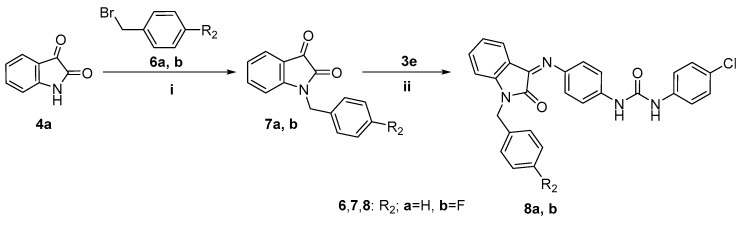
Synthesis of target compounds **8a**,**b** *Reagents and conditions*: **i**, DMF, K_2_CO_3_, reflux 2h; **ii**, EtOH, AcOH (catalytic), reflux 3 h.

The synthetic pathway adopted to synthesize the target indolinone derivatives is outlined in Scheme 1 and Scheme 2. Synthesis was initiated by reacting 4-nitrophenyl isocyanate with the appropriate anilines **1a**-**e** to afford 1-(4-nitrophenyl)-3-phenylureas **2a**–**e**, followed by hydrogenation using Pd/C in methanol to furnish the corresponding amines **3a**–**e**. The target indolinone derivatives **5a**–**x** were obtained in good yields (75%–81%) through the condensation of the amino intermediates **3a**–**e** with indole-2,3-diones **4a**–**e** in ethyl alcohol in the presence of a catalytic amount of glacial acetic acid reaction (Scheme 1).

Finally, indol-2,3-dione **4a** was refluxed with benzyl bromides **6a**,**b** in dry DMF and anhydrous K_2_CO_3_ to yield *N*-substituted indol-2,3-diones **7a**,**b**, respectively, which were condensed with the key intermediate **3e** in ethyl alcohol in the presence of a catalytic amount of glacial acetic acid to furnish the target indolinones **8a**,**b** in 77% and 83% yield, respectively (Scheme 2).

The structures of all newly synthesized derivatives were verified on the basis of spectral and elemental analyses which were in full agreement with the proposed structures.

### 2.2. Biological Evaluation

The *in vitro* cytotoxic activity of the prepared compounds **5a**–**s** and **8a**,**b** was examined against two different cell lines (MCF-7 and PC-3) utilizing the colorimetric assay (SRB) developed by Skehan *et al.* [28] using doxorubicin as a reference drug (Table 1).

As shown in Table 1, MCF-7 cells were found to be more susceptible to the synthesized compounds than prostate cancer (PC-3) cells. The majority of the tested compounds exhibited a competitive cytotoxic activity against MCF-7 cells. In particular, compounds **5l**, **5o**, **5q** and **8b** exhibited potent cytotoxic activity against MCF-7, with IC_50_ = 1.93 ± 0.17, 1.04 ± 0.10, 3.87 ± 0.31 and 4.66 ± 0.42 μM, or 3.8-, 7-, 1.88- and 1.56-fold increased activity as compared to the reference drug, doxorubicin (IC_50_ = 7.3 ± 0.84 μM), respectively. Compound **5n** possessed cytotoxic activity close to the activity of the reference drug doxorubicin, with IC_50_ = 9.60 ± 1.08 μM. Compounds **5a**–**c**, **5p**, **5r** and **8a** were also moderately active towards MCF-7 with IC_50_ values in the range of 12.16 ± 1.17–28.00 ± 2.93 μM. Other analogues showed weak or no activity against MCF-7 cell lines (Table 1).

Concerning activity against PC-3, the synthesized compounds showed moderate to low activity against this cell line. In particular, compounds **8a**,**b** were the most active analogues against PC-3 cell line through this study with IC_50_ values of 18.40 ± 1.33 and 13.79 ± 0.88 μM, respectively (Table 1).

Regarding the activity against MCF-7 cells, SAR studies provided useful conclusions about the effects of substitutions on both the indolinone moiety and the terminal phenyl group of the biphenylurea moiety. Regarding our strategy, we have four different possibilities regarding substitution depending on whether the substituent is electron donating or electron withdrawing using two different positions. Herein, electron withdrawing substituents with different types and position were added to the terminal phenyl group of the biphenylurea moiety, while variable substituents were used on the indolinone moiety. Concerning substitution on the terminal phenyl group of the biphenylurea moiety, derivatives with chloro-substituents on either position 3 or 4 produced some derivatives with better cytotoxic activity than other substituents, as exemplified by compounds **5l**, **5o**, **5q** and **8b** (Table 1). It was noticed that the substitution on indolinone moiety should be either Cl or no substitution. The other derivatives such as **5j**, **5k**, **5m**, **5n**, **5p**, **5r**, **5s** and **8a**, with showed intermediate activity or no activity as it may be affected by the 5-substituents on indolinone moiety that are either with no substitution or with electron donating substitution except compound **5p** with 5-F substitution. With no substituents on the terminal phenyl group of the biphenylurea moiety, compounds **5a**–**c** showed intermediate cytotoxic activity, while 4-F or 3-CF_3_ substitution resulted in weak or no activity as in compounds **5d**,**5i**.

Finally, exploration of the impact of the substitution on the N atom of the indolinone suggested that this was an advantageous approach for activity against PC-3, where the N-substituted members **8a** and **8b** (IC_50_ = 18.40 ± 1.33 and 13.79 ± 0.88 μM, respectively) showed enhanced activity over their unsubstituted counterpart **5o** (IC_50_ = 83.54 ± 6.32 μM) towards PC-3. Nevertheless such an approach failed to improve the activity against MCF-7 cells (Table 1).

### 2.3. Physicochemical Properties and ADME Profiling

According to Table 2, it is worthy to mention that all of the synthesized compounds (**5a**–**s** and **8a**,**b**) passed the filter of both Lipinski’s rule of five [29] and the Veber rule [30]. The previous fact suggested all the synthesized compounds possess good oral bioavailability. Additionally, several ADME descriptors for all the synthesized compounds **5a**–**s** and **8a**,**b** were estimated through a theoretical kinetic study carried out using the Discovery Studio 2.5 software (Table 3, Accelrys, San Diego, CA, USA). All of the synthesized compounds showed good predicted levels of human intestinal absorption except **8a**,**b** that showed moderate intestinal absorption. Regarding aqueous solubility, all of the tested compounds ranged from very low to low solubility. Concerning blood-brain barrier penetration, all of the tested compounds were discovered to possess medium penetrability except compounds **5i**, and **8a**,**b** that were undefined. Compounds **5f**, **5g**, **5h** and **5p** were predicted to be CYP2D non-inhibitors.

## 3. Experimental Section

### 3.1. General Information

Melting points were measured with a Stuart melting point apparatus and are uncorrected. Infrared (IR) spectra were recorded as potassium bromide disks on a FT-IR 8400S spectrophotometer (Shimadzu, Kyoto, Japan) and values are expressed in wavenumber (cm^−1^) NMR spectra were recorded on an AV-400 MHz NMR spectrophotometer (Bruker, Karlsruhe, Germany). ^1^H-NMR spectra were run at 400 MHz and ^13^C spectra were run at 100 MHz in deuterated dimethylsulfoxide (DMSO-*d_6_*). Chemical shifts are expressed in δ values (ppm) using the solvent peak as internal standard. All coupling constants (*J*) values are given in Hertz. The abbreviations used to indicate peak multiplicity are as follows: s, singlet; d, doublet; m, multiplet. Elemental analyses were carried out at the Regional Center for Microbiology and Biotechnology, Al-Azhar University, Cairo, Egypt. Reaction courses and product mixtures were routinely monitored by thin layer chromatography (TLC) on silica gel precoated F_254_ plates Merck (Merck KGaA, Darmstadt, Germany). Unless otherwise noted, all solvents and reagents were commercially available and used without further purification. Compounds **2a**–**e**, **3a**–**e**, **5a**–**l** and **5o**–**q** [4,31] and **7a**,**b** [32] were all previously prepared.

### 3.2. Synthesis

#### 3.2.1. *1-(4-Nitrophenyl)-3-phenyl/substituted phenylurea* **2a**–**e**

Compounds **2a**–**e** were prepared according to the literature procedure [3,30].

#### 3.2.2. *1-(4-Aminophenyl)-3-phenyl/substituted phenylurea* **3a**–**e**

Compounds **3a**–**e** were prepared according to the literature procedure [3,30].

#### 3.2.3. General Procedure for Preparation of Target Compounds **5a**–**x**

Indoline-2,3-diones **4a**–**e** (0.001 mol) were added to a suspension of the appropriate 1-(4-aminophenyl)-3-phenyl/substituted phenylurea **3a**–**e** (0.001 mol) in ethyl alcohol (10 mL) containing acatalytic amount of glacial acetic acid. The reaction mixture was heated under reflux for 3 h. The precipitate formed was collected by filtration while hot, washed with hot ethanol, dried and crystallized from ethanol/DMF to afford target compounds **5a**–**x**. ^1^H-NMR of **5m**, **5n**, **5p**, **5s**, and **8a**,**b** can be found in the Supplementary Materials.

*1-(3-Chlorophenyl)-3-(4-(5-methyl-2-oxoindolin-3-ylideneamino)phenyl)urea* (**5m**): Orange powder (yield 75%); m.p. > 300 °C; IR (KBr, ν cm^−1^): 3277 (NH), 1739, 1724 (2C=O); ^1^H-NMR (DMSO-*d*_6_) δ (ppm): 2.02 and 2.29 (s, 3H, CH_3_), 6.54 (s, 1H, Ar-H), 6.80 (d, *J* =7.92 Hz, 1H, Ar-H), 6.80 (d, *J* = 8.66 Hz, 2H, Ar-H), 7.01–7.43 (m, 4H, Ar-H), 7.56 (d, *J* = 8.66 Hz, 2H, Ar-H), 7.73 (s, 1H, Ar-H), 8.81 and 8.88 (s, 1H, D_2_O exchangeable, NH urea), 8.90 and 8.92 (s, 1H, D_2_O exchangeable, NH urea), 10.74 and 10.85 (s, 1H, D_2_O exchangeable, NH isatin); ^13^C-NMR (DMSO-*d*_6_, 100 MHz) δ: 21.03, 110.85, 111.75, 112.47, 115.28, 116.29, 117.92, 118.68, 119.79, 123.26, 125.25, 127.25, 129.35, 136.82, 137.33, 143.31, 145.11, 152.70, 154.99, 159.26; Anal. calcd. For C_22_H_17_ClN_4_O_2_ (404.85): C, 65.27; H, 4.23; N, 13.84; Found C, 65.11; H, 4.27; N, 13.93.

*1-(3-Chlorophenyl)-3-(4-(5-methoxy-2-oxoindolin-3-ylideneamino)phenyl)urea* (**5n**): Red powder (yield 78%); m.p. > 300 °C; IR (KBr, ν cm^−1^): 3275 (NH), 1738, 1724 (2C=O); ^1^H-NMR (DMSO-*d*_6_) δ (ppm): 3.50 and 3.77 (s, 3H, OCH_3_), 6.19 (s, 1H, Ar-H), 6.80 (d, *J* = 8.52 Hz, 1H, Ar-H), 6.97–7.44 (m, 6H, Ar-H), 7.56 (d, *J* = 8.64 Hz, 2H, Ar-H), 7.73 (s, 1H, Ar-H), 8.82 and 8.88 (s, 1H, D_2_O exchangeable, NH urea), 8.91 and 8.92 (s, 1H, D_2_O exchangeable, NH urea), 10.66 and 10.77 (s, 1H, D_2_O exchangeable, NH isatin); ^13^C-NMR (DMSO-*d*_6_, 100 MHz) δ: 55.73, 107.60, 111.95, 112.39, 116.70, 118.07, 119.14, 119.88, 121.92, 123.17, 130.86, 133.67, 137.51, 140.78, 141.74, 144.88, 152.91, 154.42, 155.50, 164.20;Anal. calcd. For C_22_H_17_ClN_4_O_3_ (420.10): C, 62.79; H, 4.07; N, 13.31; Found C, 62.95; H, 3.99; N, 13.24.

*1-(4-Chlorophenyl)-3-(4-(5-methyl-2-oxoindolin-3-ylideneamino)phenyl)urea* (**5r**): Orange powder (yield 81%); m.p. > 300 °C; IR (KBr, ν cm^−1^): 3273 (NH, NH_2_), 1739, 1720 (2C=O); ^1^H-NMR (DMSO-*d*_6_) δ (ppm): 2.01 and 2.29 (s, 3H, CH_3_), 6.55 (s, 1H, Ar-H), 6.79 (d, *J* = 7.90 Hz, 1H, Ar-H), 6.98 (d, *J* = 8.60 Hz, 2H, Ar-H), 7.13–7.44 (m, 3H, Ar-H), 7.50 (d, *J* = 8.78 Hz, 2H, Ar-H), 7.56 (d, *J* = 8.60 Hz, 2H, Ar-H), 8.78 and 8.87 (s, 1H, D_2_O exchangeable, NH urea), 8.85 (s, 1H, D_2_O exchangeable, NH urea), 10.74 and 10.85 (s, 1H, D_2_O exchangeable, NH isatin); ^13^C-NMR (DMSO-*d*_6_, 100 MHz) δ: 20.90, 111.73, 116.330, 118.49, 119.35, 119.63, 120.17, 122.19, 128.98, 130.83, 135.11, 137.63, 139.18, 144.71, 145.09, 152.95, 154.92, 164.21; Anal. calcd. For C_22_H_17_ClN_4_O_2_ (404.85): C, 65.27; H, 4.23; N, 13.84; Found C, 65.03; H, 4.32; N, 13.93.

*1-(4-Chlorophenyl)-3-(4-(5-methoxy-2-oxoindolin-3-ylideneamino)phenyl)urea* (**5s**): Red powder (yield 76%); m.p. > 300 °C; IR (KBr, ν cm^−1^): 3283 (NH), 1740, 1724 (2C=O); ^1^H-NMR (DMSO-*d*_6_) δ (ppm): 3.49 and 3.77 (s, 3H, CH_3_), 6.20 (s, 1H, Ar-H), 6.81 (d, *J* = 8.56 Hz, 1H, Ar-H), 6.95–7.16 (m, 3H, Ar-H), 7.32 (d, *J* = 8.84 Hz, 2H, Ar-H), 7.50 (d, *J* = 8.84 Hz, 2H, Ar-H), 7.57 (d, *J* = 8.72 Hz, 2H, Ar-H), 8.86 and 8.94 (s, 1H, D_2_O exchangeable, NH urea), 8.92 (s, 1H, D_2_O exchangeable, NH urea), 10.66 and 10.77 (s, 1H, D_2_O exchangeable, NH isatin); ^13^C-NMR (DMSO-*d*_6_, 100 MHz) δ: 55.71, 112.06, 112.38, 115.70, 118.47, 119.16, 120.17, 120.40, 122.28, 125.81, 129.07, 137.71, 139.22, 140.77, 144.73, 152.99, 154.46, 164.23; Anal. calcd. For C_22_H_17_ClN_4_O_3_ (420.10): C, 62.79; H, 4.07; N, 13.31; Found C, 62.95; H, 4.11; N, 13.42.

#### 3.2.4. *N-Benzylindoline-2,3-diones* **7a**,**b**

Compounds **7a**,**b** were prepared according to the literature procedure [31].

#### 3.2.5. General Procedure for Preparation of Target Compounds **8a**,**b**

*1-(4-(1-Benzyl-2-oxoindolin-3-ylideneamino)phenyl)-3-(4-chlorophenyl)urea* (**8a**): Orange powder (yield 77%); m.p. > 300 °C; IR (KBr, ν cm^−1^): 3275 (NH), 1736 (2C=O); ^1^H-NMR (DMSO-*d*_6_) δ (ppm): 4.88 and 4.99 (s, 2H, -CH_2_), 6.77–7.11 (m, 4H, Ar-H), 7.23–7.47 (m, 9H, Ar-H), 7.50 (d, *J* = 8.84 Hz, 2H, Ar-H), 7.57 (d, *J* = 8.64 Hz, 2H, Ar-H), 8.83 and 8.87 (s, 1H, D_2_O exchangeable, NH urea), 8.86 (s, 1H, D_2_O exchangeable, NH urea); ^13^C-NMR (DMSO-*d*_6_, 100 MHz) δ: 43.15, 111.08, 115.94, 118.40, 119.30, 120.20, 122.02, 123.38, 125.32, 127.83, 128.03, 129.11, 129.20, 134.52, 136.40, 137.83, 139.17, 144.64, 147.21, 152.95, 153.94, 163.00; Anal. calcd. For C_28_H_21_ClN_4_O_2_ (480.14): C, 69.92; H, 4.40; N, 11.65; Found C, 69.75; H, 4.47; N, 11.56.

*1-(4-Chlorophenyl)-3-(4-(1-(4-fluorobenzyl)-2-oxoindolin-3-ylideneamino)phenyl)urea* (**8b**): Yellow powder (yield 83%); m.p. > 300 °C; IR (KBr, ν cm^−1^): 3273 (NH), 1738, 1721 (2C=O); ^1^H-NMR (DMSO-*d*_6_) δ (ppm): 4.87 and 4.99 (s, 2H, -CH_2_), 6.77 (d, *J* = 7.56 Hz, 1H, Ar-H), 6.82 (t, *J* = 7.52 Hz,1H, Ar-H), 6.98–7.25 (m, 5H, Ar-H), 7.33–7.52 (m, 5H, Ar-H), 7.57 (d, *J* = 8.60 Hz, 2H, Ar-H), 7.64 (d, *J* = 8.36 Hz, 2H, Ar-H), 8.83 and 8.87 (s, 1H, D_2_O exchangeable, NH urea), 8.86 (s, 1H, D_2_O exchangeable, NH urea); ^13^C-NMR (DMSO-*d*_6_, 100 MHz) δ: 42.62, 111.02, 115.87, 116.08, 119.31, 119.66, 120.23, 122.92, 125.33, 125.86, 129.10, 129.97, 133.89, 137.84, 139.16, 144.62, 147.05, 152.95, 153.90, 157.60, 160.82, 163.00; Anal. calcd. For C_28_H_20_ClFN_4_O_2_ (498.94): C, 67.40; H, 4.04; N, 11.23; Found C, 67.59; H, 4.11; N, 11.32.

### 3.3. In Vitro Cytotoxic Activity

#### 3.3.1. Cell Culture

Human breast cancer (MCF-7) and prostate cancer (PC-3) cells were purchased from National Cancer Institute (Cairo, Egypt). Maintenance of cells was carried out using Roswell Park Memorial Institute medium (RPMI-1640) supplemented with 10% heat-inactivated fetal bovine serum, 100 Units/mL of penicillin and 100 μg/mL streptomycin. Cells were kept as a monolayer culture by serial passaging under standard culture conditions of 37 °C, 95% humidified air and 5% CO_2_.

#### 3.3.2. *In vitro* Cytotoxic Activity

Investigated compounds were dissolved in DMSO and kept at a stock concentration of 100 mM. Cytotoxicity was assessed via SRB method as indicated by Skehan *et al.* [28] In brief, exponentially growing cells were collected using 0.25% Trypsin-EDTA and seeded in 96-well plates at a 3000 cells/well density in culture medium and incubated for 24 h till the formation of a monolayer. Subsequently, cells were incubated with different concentrations (0–10^3^ μM) of the compounds under investigation as well as doxorubicin as a reference drug for further 72 h. At the end of the incubation, the treatment was terminated through fixing the cells with 10% trichloroacetic acid for 1 h at 4 °C. This was followed by a staining procedure using 0.4% SRB (1% acetic acid) for 10 min at room temperature. Finally, microplates were left to dry for 24 h and Tris-HCl was added afterwards for 5 min on a shaker at 1600 rpm for solubilization. Using an ELISA microplate reader (ChroMate-4300, Palm City, FL, USA), optical density was determined at 545 nm. The IC_50_ values were calculated according to the equation for Boltzman sigmoidal concentration–response curve using the nonlinear regression fitting models (Graph Pad Prism Version 5; GraphPad Software, Inc. La Jolla, CA, USA). The results reported are means of at least three separate experiments. Statistical analyses were carried out using one-way analysis of variance (ANOVA) followed by the Tukey-Kramer test for *post-hoc* analysis. Statistical difference was considered to be significant at *p* < 0.05. Statistical testing was performed using GraphPad InStat software, version 3.05.

#### 3.3.3. Physicochemical Properties and ADME Profiling

Physicochemical properties and ADME profiling for all the synthesized compounds were performed using Discovery Studio 4 (Accelrys, San Diego, CA, USA). All the tested compounds were drawn as a small library and prepared using prepare ligand protocol to find the suitable orientation in 3D. For studying physicochemical properties, the prepared library was filtered using the Lipinski and Veber rules protocols. ADME profiling was predicted for the designed library using ADME descriptors protocol.

## 4. Conclusions

We reported herein the synthesis, *in vitro* cytotoxic activity and *in silico* ADME prediction studies of twenty one biphenylurea derivatives **5a**–**s** and **8a**,**b** that incorporated an indolinone moiety. All the synthesized compounds were evaluated for their *in vitro* cytotoxicity against two human cancer cell lines, namely MCF-7 breast cancer and PC-3 prostate cancer. Compounds **5l**, **5o**, **5q** and **8b** exhibited potent cytotoxic activity against MCF-7 with IC_50_ = 1.93, 1.04, 3.87 and 4.66 μM, respectively and all of these compounds were found to be more active than the reference drug doxorubicin (IC_50_ = 7.3 μM). All of the tested compounds were filtered according to the Lipinski and Veber rules and all of them passed the filter. In addition they showed varied physicochemical properties according to their ADME descriptors.

## Figures and Tables

**Table 1:** *In vitro* cytotoxic activities of the synthesized derivatives against MCF-7 and PC-3 cell lines. 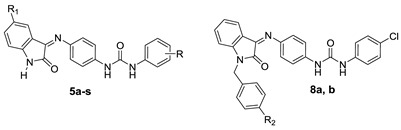

Compd.	R	R_1_	R_2_	IC_50_ (μM) ^a^	
				MCF-7	PC-3
**5a**	H	H	-	19.53 ± 1.05	NA ^b^
**5b**	H	F	-	17.14 ± 0.66	NA ^b^
**5c**	H	Cl	-	19.17 ± 1.94	20.43 ± 1.51
**5d**	4-F	H	-	NA ^b^	NA ^b^
**5e**	4-F	F	-	NA ^b^	NA ^b^
**5f**	4-F	Cl	-	48.99 ± 3.74	NA ^b^
**5g**	3-CF_3_	H	-	39.53 ± 4.02	NA ^b^
**5h**	3-CF_3_	F	-	NA ^b^	51.76 ± 4.01
**5i**	3-CF_3_	Cl	-	97.65 ± 7.39	NA ^b^
**5j**	3-Cl	H	-	NA ^b^	NA ^b^
**5k**	3-Cl	F	-	75.92 ± 7.21	NA ^b^
**5l**	3-Cl	Cl	-	1.93 ± 0.17	NA ^b^
**5m**	3-Cl	Me	-	35.59 ± 3.27	73.22 ± 5.07
**5n**	3-Cl	OMe	-	9.60 ± 1.08	96.00 ± 6.85
**5o**	4-Cl	H	-	1.04 ± 0.10	83.54 ± 6.32
**5p**	4-Cl	F	-	26.02 ± 2.51	NA ^b^
**5q**	4-Cl	Cl	-	3.87 ± 0.31	NA ^b^
**5r**	4-Cl	Me	-	12.16 ± 1.17	NA ^b^
**5s**	4-Cl	OMe	-	43.08 ± 4.22	97.00 ± 3.25
**8a**	-	-	H	28.00 ± 2.93	18.40 ± 1.33
**8b**	-	-	F	4.66 ± 0.42	13.79 ± 0.88
Dox.	-	-	-	7.30 ± 0.84	0.84 ± 0.079

^a^ IC_50_ values are presented as mean ± S.D. of three separate experiments; ^b^ NA: Compounds having IC_50_ value > 100 μM.

**Table 2:** Lipinski rule of five parameters results for all the synthesized compounds.

Compd.	H-bond Donor ^a,#^	H-bond Acceptor ^b,#^	Molecular Weight	AlogP ^d^	No. of Rotatable Bonds ^e^	Polar Surface Area ^f^ (Å)
**5a**	3	6	356.4	3.200	3	82.59
**5b**	3	6	374.4	3.405	3	82.59
**5c**	3	6	390.8	3.864	3	82.59
**5d**	3	6	374.4	3.405	3	82.59
**5e**	3	6	392.4	3.611	3	82.59
**5f**	3	6	408.8	4.07	3	82.59
**5g**	3	6	424.4	4.142	4	82.59
**5h**	3	6	442.4	4.348	4	82.59
**5i**	3	6	458.8	4.807	4	82.59
**5j**	3	6	390.8	3.864	3	82.59
**5k**	3	6	408.8	4.07	3	82.59
**5l**	3	6	425.3	4.529	3	82.59
**5m**	3	6	404.8	4.351	3	82.59
**5n**	3	7	420.8	3.848	4	91.82
**5o**	3	6	390.8	3.864	3	82.59
**5p**	3	6	408.8	4.07	3	82.59
**5q**	3	6	425.3	4.529	3	82.59
**5r**	3	6	404.8	4.351	3	82.59
**5s**	3	7	420.8	3.848	4	91.82
**8a**	2	6	480.9	5.654 *	5	73.8
**8b**	2	6	498.9	5.859 *	5	73.8

**^a^** The upper limit of the number of hydrogen bond donors is 5; ^b^ The upper limit of the number of hydrogen bond acceptors is 10; ^c^ The upper limit of the molecular weight is 500; ^d^ The upper limit of the AlogP (the log value of octanol-water partition coefficient) is 500; ^e^ The upper limit of the number of rotatable bonds is 10; * No. of violation allowed is 1 and AlogP value should not be ≥ 6; ^f^ The upper limit of the polar surface area is 140 Å; ^#^ The upper limit of the sum of hydrogen bond donor and acceptors is 12.

**Table 3:** ADME studies results for all the synthesized compounds.

Compd.	ADMET Absorption Level ^a^	ADMET Solubility ^b^	ADMET Solubility Level ^c^	ADMET BBB ^d^	ADMET BBB Level ^d^	CYP2D6 Probability	CYP2D6 ^e^
**5a**	0	−4.438	2	−0.500	2	0.722	1
**5b**	0	−4.899	2	−0.436	2	0.594	1
**5c**	0	−5.299	2	−0.294	2	0.584	1
**5d**	0	−4.878	2	−0.436	2	0.633	1
**5e**	0	−5.331	2	−0.373	2	0.584	1
**5f**	0	−5.732	2	−0.231	2	0.485	0
**5g**	0	−6.048	1	−0.208	2	0.495	0
**5h**	0	−6.477	1	−0.145	2	0.465	0
**5i**	0	−6.877	1	NA	4	0.544	1
**5j**	0	−5.289	2	−0.294	2	0.683	1
**5k**	0	−5.742	2	−0.231	2	0.504	1
**5l**	0	−6.143	1	−0.089	2	0.514	1
**5m**	0	−5.734	2	−0.144	2	0.653	1
**5n**	0	−5.415	2	−0.441	2	0.683	1
**5o**	0	−5.279	2	−0.294	2	0.623	1
**5p**	0	−5.732	2	−0.231	2	0.485	0
**5q**	0	−6.132	1	−0.089	2	0.504	1
**5r**	0	−5.723	2	−0.144	2	0.643	1
**5s**	0	−5.404	2	−0.441	2	0.524	1
**8a**	1	-6.436	1	NA	4	0.712	1
**8b**	1	-6.737	1	NA	4	0.762	1

^a^ Human intestinal absorption level. (0 = good, 1 = moderate, 2 = poor, 3 = very poor); ^b^ The base 10 logarithm of the molar solubility as predicted by the regression model based by DS; ^c^ Categorical solubility level. (0 = extreme low, 1 = very low but possible, 2 = low, 3 = good, 4 = optimal); ^d^ Blood brain barrier penetration. (0; ≥ 0.7 = very high penetrant, 1; 0 ≤ logBB < 0.7) = High penetrant, 2; −0.52 < LogBB < 0 = low, 3; logBB ≤ −0.52 = low, 4; NA = undefiend); ^e^ CYP2D inhibition. (0 = non inhibitor, 1 = inhibitor).

## Supplementary Materials

Supplementary materials can be accessed at: http://www.mdpi.com/1420-3049/21/6/762/s1.

## References

[1] World Cancer Report 2014. http://www.iarc.fr/en/publications/books/wcr/wcr-order.php.

[2] Torre L.A., Bray F., Siegel R.L., Ferlay J., Lortet-Tieulent J., Jemal A. (2015). Global cancer statistics, 2012. CA-Cancer J. Clin..

[3] Havrylyuk D., Kovach N., Zimenkovsky B., Vasylenko O., Lesyk R. (2011). Synthesis and Anticancer Activity of Isatin-Based Pyrazolines and Thiazolidines Conjugates. Arch. Pharm..

[4] Eldehna W.M., Fares M., Ibrahim H.S., Aly M.H., Zada S., Ali M.M., Abou-Seri S.M., Abdel-Aziz H.A., El Ella D.A.A. (2015). Indoline ureas as potential anti-hepatocellular carcinoma agents targeting VEGFR-2: Synthesis, *in vitro* biological evaluation and molecular docking. Eur. J. Med. Chem..

[5] Eldehna W.M., Ibrahim H.S., Abdel-Aziz H.A., Farrag N.N., Youssef M.M. (2015). Design, synthesis and *in vitro* antitumor activity of novel *N*-substituted-4-phenyl/benzylphthalazin-1-ones. Eur. J. Med. Chem..

[6] Ibrahim H.S., Abou-seri S.M., Ismail N.S., Elaasser M.M., Aly M.H., Abdel-Aziz H.A. (2016). Bis-isatin hydrazones with novel linkers: Synthesis and biological evaluation as cytotoxic agents. Eur. J. Med. Chem..

[7] Sławiński J., Grzonek A., Żołnowska B., Kawiak A. (2015). Synthesis of Novel Pyrido[4,3-*e*][1,2,4] triazino[3,2-*c*][1,2,4]thiadiazine 6,6-dioxide Derivatives with Potential Anticancer Activity. Molecules.

[8] Fares M., Eldehna W.M., Abou-Seri S.M., Abdel-Aziz H.A., Aly M.H., Tolba M.F. (2015). Design, Synthesis and *in Vitro* Antiproliferative Activity of Novel Isatin-Quinazoline Hybrids. Arch. Pharm..

[9] Wilhelm S., Carter C., Lynch M., Lowinger T., Dumas J., Smith R.A., Schwartz B., Simantov R., Kelley S. (2006). Discovery and development of sorafenib: a multikinase inhibitor for treating cancer. Nat. Rev. Drug Discov..

[10] Woo H.Y., Heo J. (2012). Sorafenib in liver cancer. Expert. Opin. Pharmacother..

[11] Wilhelm S., Dumas J., Ladouceur G., Lynch M., Scott W. (2007). Diaryl Ureas with Kinase Inhibiting Activity. U.S. Patent.

[12] Wilhelm S.M., Dumas J., Adnane L., Lynch M., Carter C.A., Schutz G., Thierauch K.H., Zopf D. (2011). Regorafenib (BAY 73–4506): A new oral multikinase inhibitor of angiogenic, stromal and oncogenic receptor tyrosine kinases with potent preclinical antitumor activity. Int. J. Cancer.

[13] DiGiulio S. (2013). FDA Approves Stivarga for Advanced GIST. Oncol. Times.

[14] Tan E.-H., Goss G.D., Salgia R., Besse B., Gandara D.R., Hanna N.H., Yang J.C.-H., Thertulien R., Wertheim M., Mazieres J. (2011). Phase 2 trial of Linifanib (ABT-869) in patients with advanced non-small cell lung cancer. J. Thorac. Oncol..

[15] Supuran C.T., Winum J.-Y. (2015). Designing carbonic anhydrase inhibitors for the treatment of breast cancer. Expert Opin. Drug Discov..

[16] Lomelino C.L., Mahon B.P., McKenna R., Carta F., Supuran C.T. (2016). Kinetic and X-ray crystallographic investigations on carbonic anhydrase isoforms I, II, IX and XII of a thioureido analog of SLC-0111. Bioorg. Med. Chem..

[17] Vine K., Matesic L., Locke J., Ranson M., Skropeta D. (2009). Cytotoxic and anticancer activities of isatin and its derivatives: a comprehensive review from 2000–2008. Anti-Cancer Agents Med. Chem..

[18] Vine K.L., Matesic L., Locke J.M., Skropeta D. (2013). Recent highlights in the development of isatin-based anticancer agents. Adv. Anticancer Agents Med. Chem..

[19] Motzer R.J., Michaelson M.D., Redman B.G., Hudes G.R., Wilding G., Figlin R.A., Ginsberg M.S., Kim S.T., Baum C.M., DePrimo S.E. (2006). Activity of SU11248, a multitargeted inhibitor of vascular endothelial growth factor receptor and platelet-derived growth factor receptor, in patients with metastatic renal cell carcinoma. J. Clin. Oncol..

[20] Prenen H., Cools J., Mentens N., Folens C., Sciot R., Schöffski P., Van Oosterom A., Marynen P., Debiec-Rychter M. (2006). Efficacy of the kinase inhibitor SU11248 against gastrointestinal stromal tumor mutants refractory to imatinib mesylate. Clin. Cancer Res..

[21] London C.A., Malpas P.B., Wood-Follis S.L., Boucher J.F., Rusk A.W., Rosenberg M.P., Henry C.J., Mitchener K.L., Klein M.K., Hintermeister J.G. (2009). Multi-center, placebo-controlled, double-blind, randomized study of oral toceranib phosphate (SU11654), a receptor tyrosine kinase inhibitor, for the treatment of dogs with recurrent (either local or distant) mast cell tumor following surgical excision. Clin. Cancer Res..

[22] Pandit B., Sun Y., Chen P., Sackett D.L., Hu Z., Rich W., Li C., Lewis A., Schaefer K., Li P.-K. (2006). Structure–activity-relationship studies of conformationally restricted analogs of combretastatin A-4 derived from SU5416. Bioorg. Med. Chem..

[23] Griffith R., Brown M., McCluskey A., Ashman L. (2006). Small molecule inhibitors of protein kinases in cancer-how to overcome resistance. Mini Rev. Med. Chem..

[24] Sessa C., Viganò L., Grasselli G., Trigo J., Marimon I., Lladò A., Locatelli A., Ielmini N., Marsoni S., Gianni L. (2006). Phase I clinical and pharmacological evaluation of the multi-tyrosine kinase inhibitor SU006668 by chronic oral dosing. Eur. J. Cancer.

[25] Laird A.D., Vajkoczy P., Shawver L.K., Thurnher A., Liang C., Mohammadi M., Schlessinger J., Ullrich A., Hubbard S.R., Blake R.A. (2000). SU6668 is a potent antiangiogenic and antitumor agent that induces regression of established tumors. Cancer Res..

[26] Spiekermann K., Faber F., Voswinckel R., Hiddemann W. (2002). The protein tyrosine kinase inhibitor SU5614 inhibits VEGF-induced endothelial cell sprouting and induces growth arrest and apoptosis by inhibition of c-kit in AML cells. Exp. Hematol..

[27] Eldehna W.M., Fares M., Ceruso M., Ghabbour H.A., Abou-Seri S.M., Abdel-Aziz H.A., El Ella D.A.A., Supuran C.T. (2016). Amido/ureidosubstituted benzenesulfonamides-isatin conjugates as low nanomolar/subnanomolar inhibitors of the tumor-associated carbonic anhydrase isoform XII. Eur. J. Med. Chem..

[28] Skehan P., Storeng R., Scudiero D., Monks A., McMahon J., Vistica D., Warren J.T., Bokesch H., Kenney S., Boyd M.R. (1990). New colorimetric cytotoxicity assay for anticancer-drug screening. J. Natl. Cancer. Inst..

[29] Lipinski C.A., Lombardo F., Dominy B.W., Feeney P.J. (2012). Experimental and computational approaches to estimate solubility and permeability in drug discovery and development settings. Adv. Drug Deliv. Rev..

[30] Veber D.F., Johnson S.R., Cheng H.-Y., Smith B.R., Ward K.W., Kopple K.D. (2002). Molecular properties that influence the oral bioavailability of drug candidates. J. Med. Chem..

[31] Eldehna W.M., Abou-Seri S.M., El Kerdawy A.M., Ayyad R.R., Hamdy A.M., Ghabbour H.A., Ali M.M., El Ella D.A.A. (2016). Increasing the binding affinity of VEGFR-2 inhibitors by extending their hydrophobic interaction with the active site: Design, synthesis and biological evaluation of 1-substituted-4-(4-methoxybenzyl) phthalazine derivatives. Eur. J. Med. Chem..

[32] Presset M., Mohanan K., Hamann M., Coquerel Y., Rodriguez J. (2011). 1, 3-Dipolar cycloaddition of hydrazones with α-oxo-ketenes: A three-component stereoselective entry to pyrazolidinones and an original class of spirooxindoles. Org. Lett..

