# Supplementary material for: Synthesis and Cytotoxic Activity of Biphenylurea Derivatives Containing Indolin-2-one Moieties
Source: Molecules. 2016 Jun 10;21(6):762. doi: 10.3390/molecules21060762 (PMC6274071; doi:10.3390/molecules21060762)
Supplement: Supplementary file 1 [file molecules-21-00762-s001.pdf]

Supplementary Materials: Synthesis and Cytotoxic Activity of Biphenylurea Derivatives Containing Indolin-2-one Moieties

Wagdy M. Eldehna, Mohamed Fares, Hany S. Ibrahim, Muhammad A. Alsherbiny, Mohamed H. Aly, Hazem A. Ghabbour and Hatem A. Abdel-Aziz

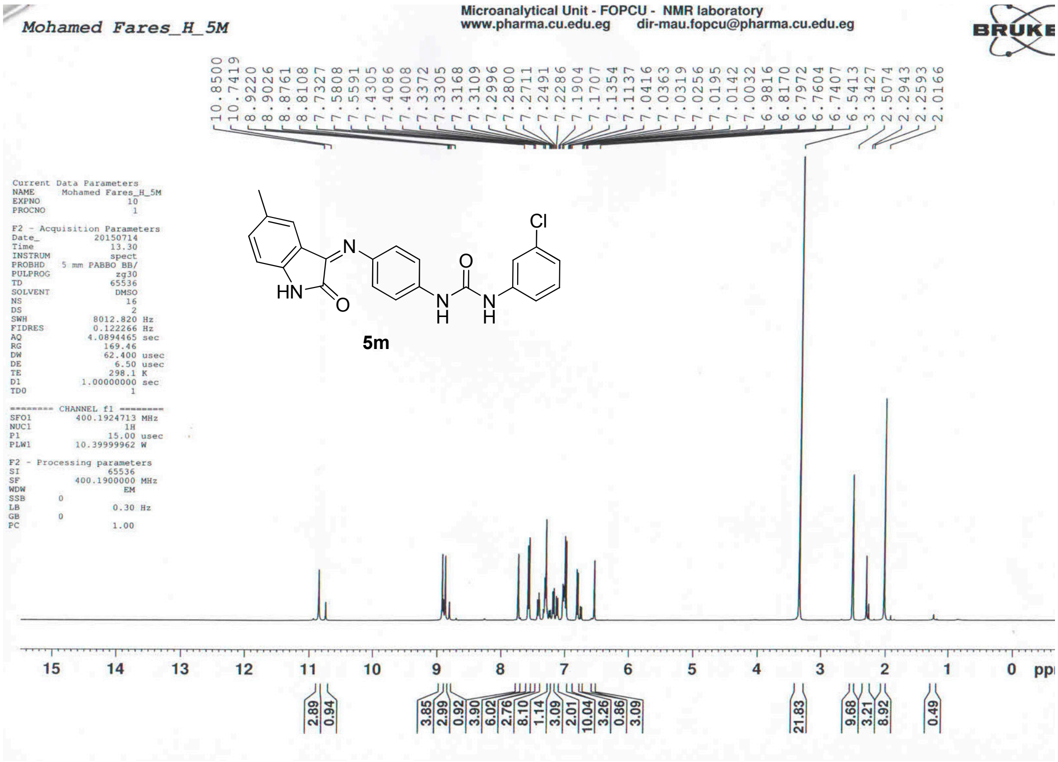

Figure S1. ^{1}H NMR spectrum of **5m**.

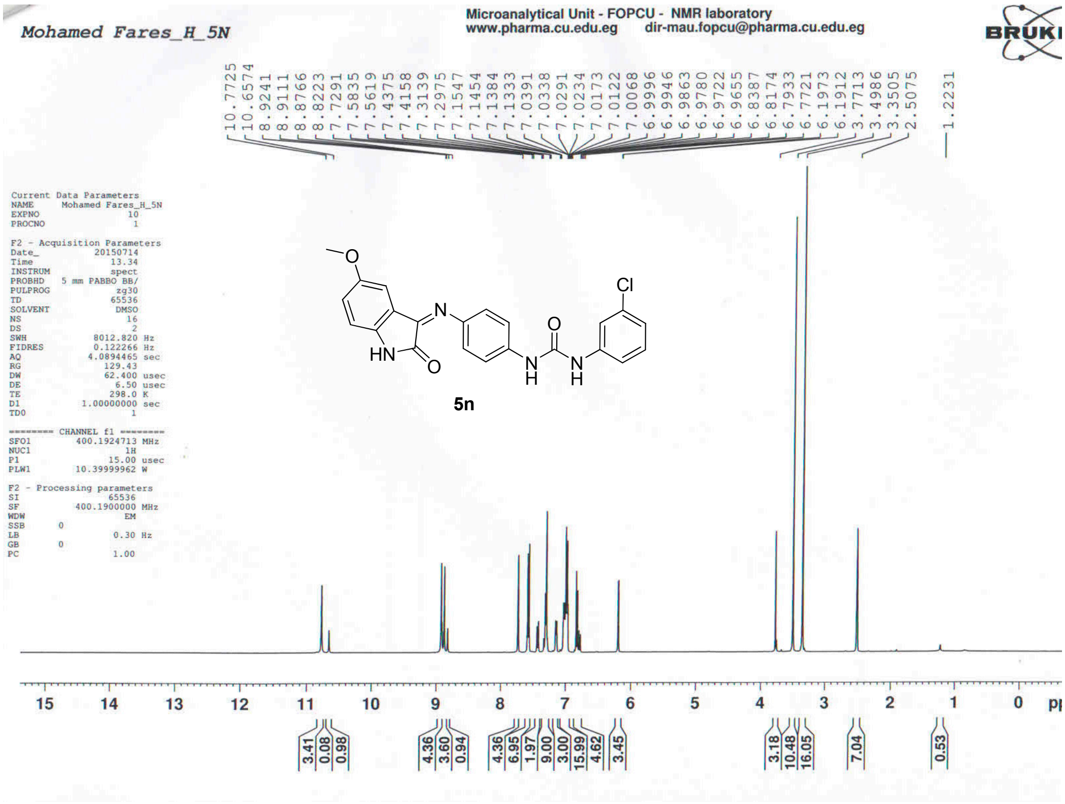

Figure S2. ^{1}H NMR spectrum of **5n**.

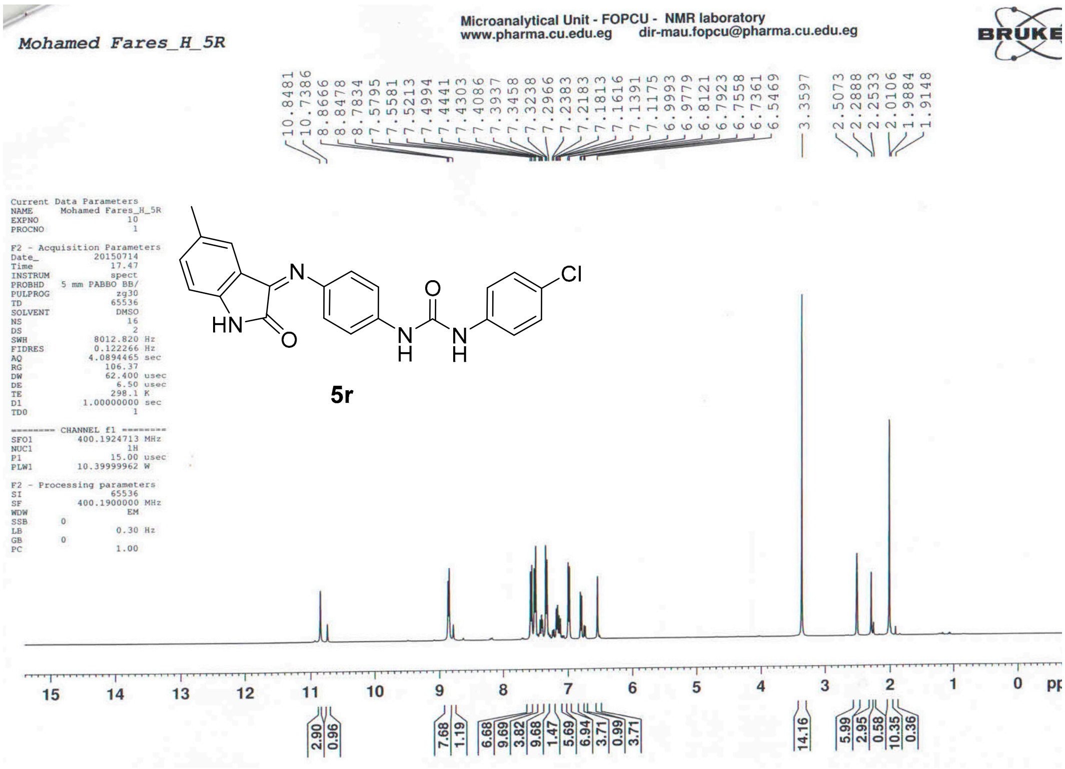

Figure S3. ^{1}H NMR spectrum of **5r**.

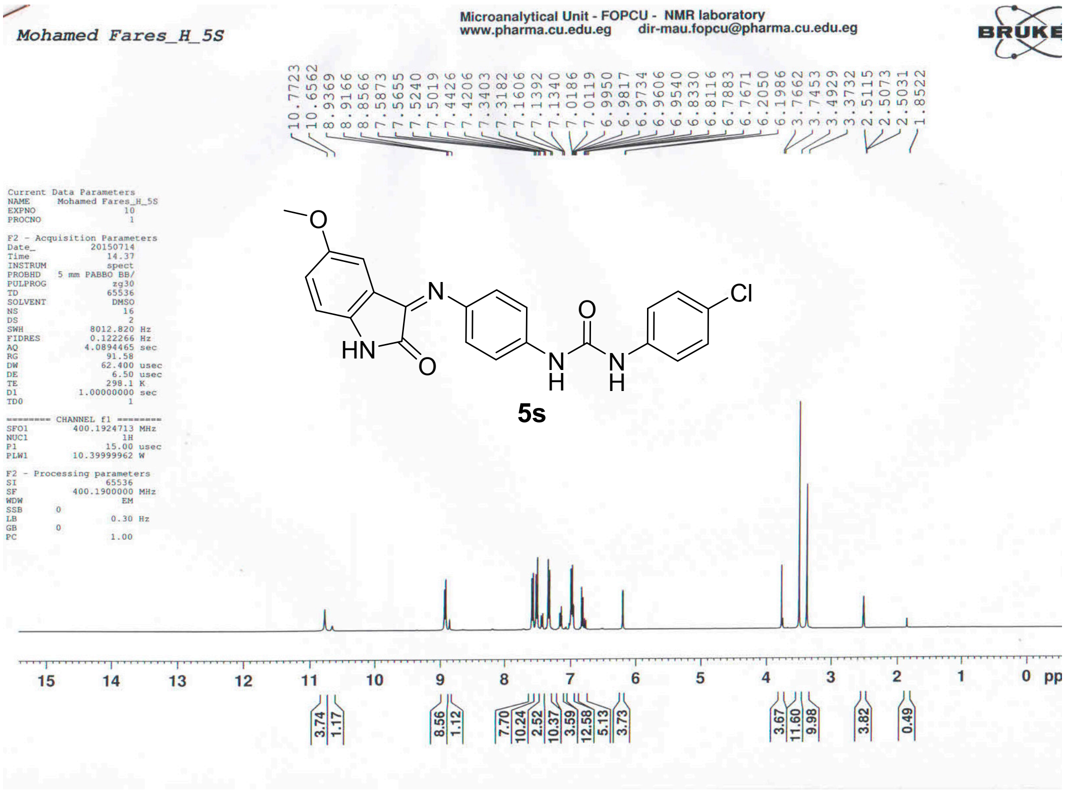

Figure S4. ^{1}H NMR spectrum of **5s.**

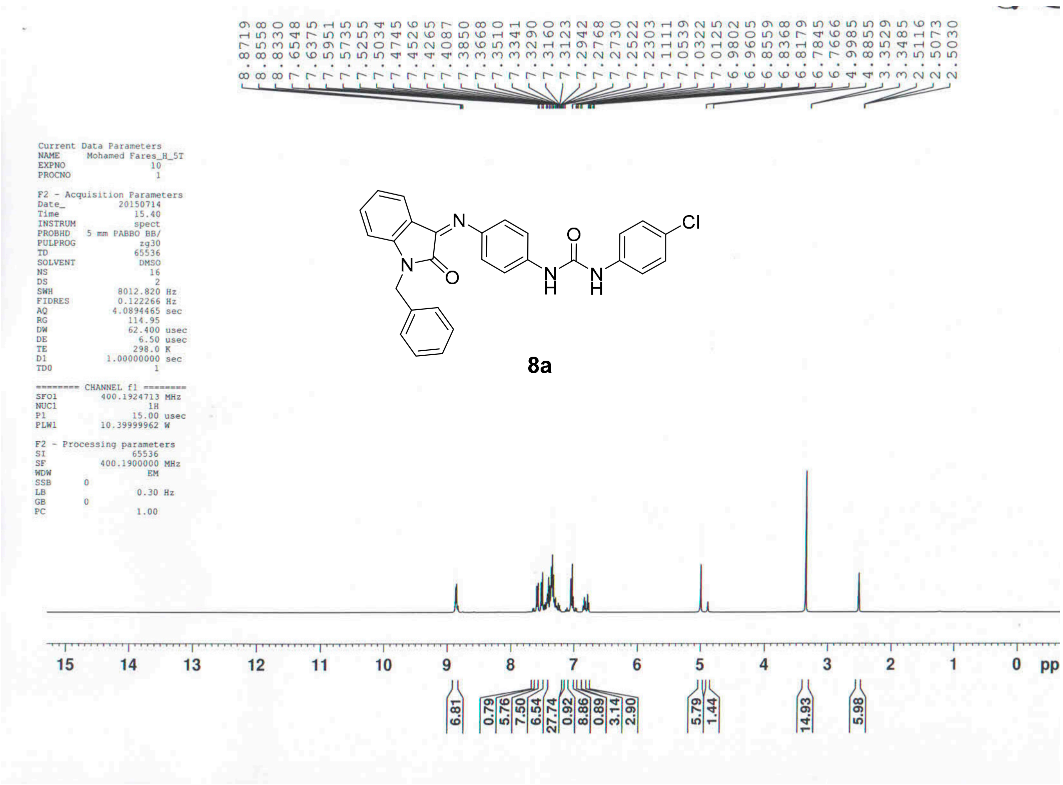

Figure S5. ^{1}H NMR spectrum of **8a.**

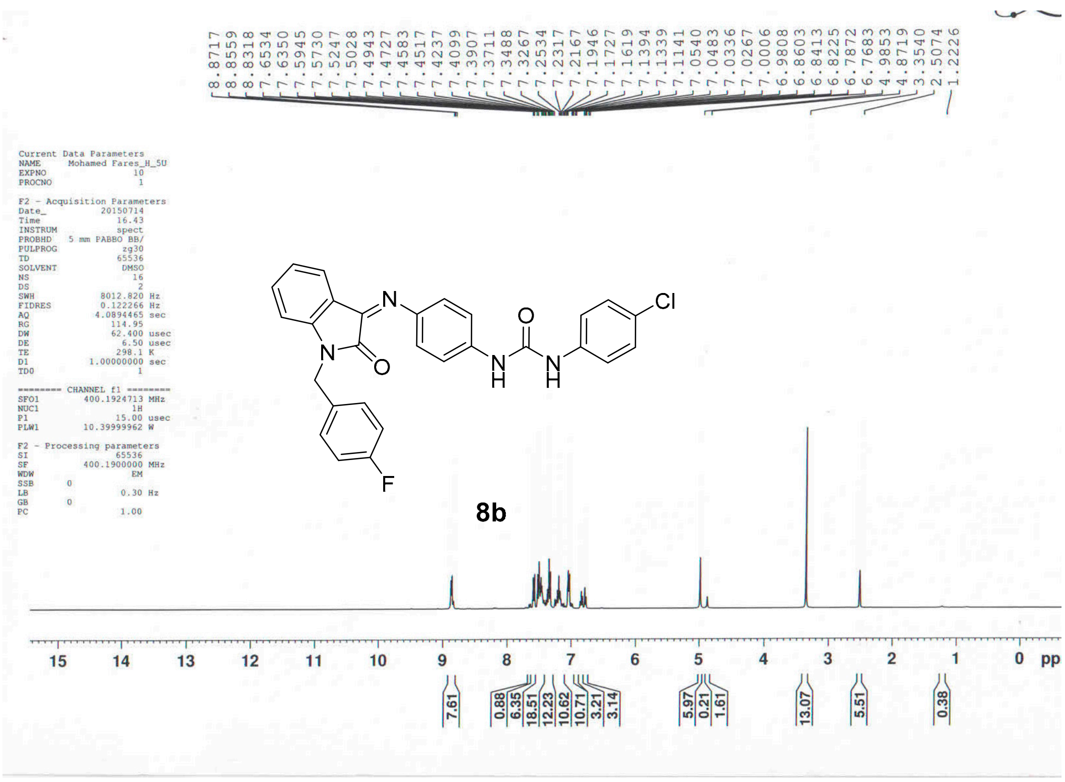

Figure S6. ^{1}H NMR spectrum of **8b.**
